# Development of a new method to quantify filler dispersion in bituminous mastics by transmission microscopy and image analysis

**DOI:** 10.1038/s41598-025-18535-4

**Published:** 2025-10-06

**Authors:** Marouane Ouazri, Walid Maherzi, Didier Lesueur, Lahcen Khouchaf

**Affiliations:** 1 IMT Nord Europe, Centre for Materials and Processes, Institut Mines-Télécom, Lille, 59000 France; 2 Western Research Institute, Laramie, WY USA

**Keywords:** Fillers, Dispersion, Agglomeration, Optical microscopy, Particle size distribution, Naphthenic and paraffinic bitumen, Engineering, Materials science

## Abstract

This study introduces a novel method to analyze how fillers disperse in bituminous binders. A custom-designed laboratory setup enables precise temperature control (140 °C) and mixing speeds (200, 800, 1500 rpm) for small-scale mixtures (5% filler) over 5, 15 and 30 min. To assess dispersion, microscopy-based visualization approach using optical microscopy (10× objective) and image analysis (ImageJ). Each particle is segmented and its projected area converted into an equivalent diameter, yielding a surface-fraction distribution (in logarithmic size bins). We evaluate the D_50_ (Area50) position and the 50–100% range to detect coarse aggregates or over-shearing. The interest of the method is illustrated by comparing how limestone (CaCO_2_) and quartz (SiO_2_) fillers disperse in two different bitumens: naphthenic and paraffinic. Results indicate that quartz disperses more readily, quickly approaching a near-homogeneous state, whereas limestone requires higher shear and longer mixing times to approach the reference filler. Paraffinic bitumen shows a higher initial fine‑particle fraction; nonetheless, the naphthenic binder de‑agglomerates faster and ultimately matches, or slightly surpasses, the paraffinic dispersion. Mixing at 1500 rpm can fracture particles, skewing the size profile. The protocol delivers ± 1% repeatability across replicates, providing a robust tool for optimising mastic formulation and enhancing pavement durability.

## Introduction

Optimizing the physical and mechanical properties of bituminous materials, used mainly in road infrastructures, is a major focus of research into civil engineering materials. For this purpose, the choice of bituminous material constituents (aggregates, fillers and bitumen), and control of the interactions between them, are essential to ensure the asphalt material’s mechanical performance and durability^1–3^. Mineral fillers (limestone and quartz) are essential ingredients in bituminous material formulations (asphalt, pavements), as they significantly influence the final performance of asphalt mixtures, in particular the rheological properties of bitumen to form the so-called mastic^4–7^. One of the major challenges in the formulation of asphalt mixtures is to ensure homogeneous dispersion of fillers in bituminous sealants. This has a direct impact on the performance and durability of the final material (pavements, waterproofing membranes or other bitumen-based systems). Even though bituminous mixtures typically contain between 3% and 10% fillers—and up to 12% in stone‑mastic asphalt (SMA), around 6% in standard semi-dense asphalt concrete conforming to EN 13,108‑1, and as much as 20–50% in fully-filled waterproofing asphalts—the uniform dispersion of filler within the bitumen remains crucial to ensuring the integrity of the mastic, which binds aggregates and contributes to the overall structural strength of the pavement. Indeed, the use of fillers is essential in bituminous materials, as they thicken the bitumen film and increase the rigidity of the mix. However, their agglomeration can lead to several negative consequences: the formation of zones of weakness due to heterogeneity, the emergence of potential breaking points, reduced durability, increased sensitivity to water and lower fatigue resistance. Indeed, this heterogeneous distribution of fillers creates fragile zones, likely to concentrate mechanical and thermal stresses, thus facilitating the formation and/or amplification of cracks over time. In addition, the rheology of the mixture may be altered, making the material less workable during application, requiring higher application temperatures. Conversely, a good dispersion of fillers reinforces the cohesion of the mix, improves the mechanical strength of the mastic and contributes to the long-term durability of the pavement. Consequently, although cracking or deterioration in bituminous structures often stems from multiple parameters (bitumen properties, climatic conditions, loading, etc.), ensuring homogeneous filler dispersion remains an essential lever for strengthening the robustness and durability of bitumen-based solutions.

Even if bitumen filler dispersion is clearly a key factor affecting asphalt mixture durability, the quality of dispersion of the filler in the bitumen has seldom been studied. So, this study was developed in order to bridge this gap and develop a method that has the potential to be used for any filler, including organic, in any bitumen. To illustrate the potential of the method, two binders and two fillers were chosen. More precisely, a naphthenic and a paraffinic bitumen were used, in combination with limestone and quartz fillers. These materials were chosen because of their known differences in behavior, as further explained below.

Fillers are known to have an impact on binder viscosity and, consequently, on the mechanical properties of mixes^8,9^ in bituminous material formulations limestone filler is often preferred^10,11^. The main role of limestone filler in bituminous mixes is to ensure greater resistance to moisture damage^11^. Limestone filler is considered the filler of reference, particularly in mastic asphalt formulations^12^.

Silicon dioxide fillers (quartz fillers) are another type of filler, used mainly in mastic asphalt mixtures, generally derived from the sandy fraction of the aggregate^13^. Due to its chemical nature and molecular structure^14^, quartz has higher thermal and chemical resistance than limestone fillers. However, siliceous aggregates are generally considered less favorable for interaction with bitumen^15–18^. This is because bitumen’s polar compounds, mainly acids, and to a lesser extent amine, are concentrated on the mineral’s surface^9^. In the case of quartz, hydrogen bonds are formed between these compounds and the mineral, which are easily displaced in the presence of water. Subsequently, insoluble salts are formed between bitumen acids and calcium ions, making the interface less sensitive to water^10,15–17^. Studies on the interaction mechanisms between fillers and bitumen^15–19^ have shown that the siloxane groups of quartz have a greater ability to form hydrogen bonds than the carbonate groups of limestone fillers. This difference is attributed to the inherent chemical structure and polarity of the siloxane groups^20^. Siloxane groups (Si-O-Si) in quartz have a significant ability to form hydrogen bonds due to the presence of highly polar Si-O bonds, which can interact effectively with polar molecules present in bitumen. In contrast, the carbonate groups (CO_3_^2–^) of calcium carbonate form mainly ionic bonds that are less effective in establishing the strong interactions necessary for hydrogen bonding with the polar components of bitumen. This greater tendency for hydrogen bonding by siloxane groups improves the compatibility and stability of the bitumen-quartz mixture, which can lead to better performance in asphalt applications.

Besides optical microscopy, other technics have been used to observe bitumen and the morphology of various mineral and additives dispersed in it. This mostly includes UV-fluorescence microscopy, Confocal Laser Scanning Microscopy (CLSM), Atomic Force Microscopy (AFM) and Scanning Electron Microscopy (SEM) in reflexion or transmission (TEM) modes. UV-fluorescence microscopy is relevant only when one of the phases fluoresces. This is for example the case of polymer-modified binders^21–23^. In the absence of polymer however, bitumen fluorescence is very limited, and this technique is therefore of little use for neat bitumen. Similarly, CLSM needs some fluorescence contrast to observe asphalt binders. In addition to polymer-modified binders^24^, it allows observing wax crystals in bitumen, whether naturally present or obtained from additivation^25^. AFM has been used for almost 30 years now to study bitumen^26^. It is however very tedious in terms of sample preparation. In addition, the interpretation of the surface texture of neat asphalt binders is still debated, even if there is a growing consensus that the so-called bee structures are due to wax crystallization^27,28^. Because of these limitations, AFM can’t be used to routinely study fillers or any additive in bituminous mastics. SEM and TEM are also used on bituminous binders. This is an extremely powerful method, but it requires heavy sample preparation and long observation time^29,30^. Just like AFM, it can’t be routinely used and was therefore not considered for this part of the study. Overall, and in agreement with the recent conclusions of a comparative study of the various microscopic techniques being used for bituminous binders^31^, optical microscopy was considered to be the most relevant technique to systematically study the dispersion of mineral fillers in bitumen; it requires only limited sample preparation and can thus be done easily at various stages of the blending process as shown in the rest of this article. Moreover, it is relatively cheap and could thus be implemented in most asphalt laboratories with limited investment.

From an experimental viewpoint, the dispersion behavior of two chemically distinct fillers, calcareous (F.C) and siliceous (F.S), is systematically investigated in two types of bitumens, naphthenic and paraffinic. The effects of specific preparation parameters, notably filler concentration (fixed at 5 wt%), mixing duration (5, 15, and 30 min), and mixing speed (200, 800, and 1500 rpm), are assessed in detail to identify clearly the optimal conditions ensuring efficient and dispersion of these fillers within bituminous mastics^32^.

Here we close that gap by imaging thin hot-mastic films in transmitted light to reveal filler particle silhouettes inside a nominally opaque matrix, then using a transparent, open-source pipeline (ImageJ: scale calibration → threshold/segmentation → Analyze Particles) to compute surface-area fractions in logarithmic size classes and follow their time-evolution during mixing. Our working hypothesis is straightforward: effective dispersion yields a surface-transfer from coarse to fine classes (e.g., Surface fraction [5.62–10] µm and Cumulative < 10 μm rise with mixing time), which our metrics capture with good repeatability (independent repeats; bias and 95% limits of agreement reported via Bland–Altman analysis).The 2 binders × 2 fillers design is not meant to generalize chemistry effects, it is used to stress-test generality and measurement robustness across distinct binder–filler chemistries. Compared with heavier SEM/AFM routes, the approach is low-cost, scriptable and fast (amenable to lab routine), while adhering to best practice in particle image analysis (scale calibration, defensible segmentation, counting).

## Materials and methods

### Bitumens

In this study two types of bitumens were used:

- A naphthenic bitumen with penetration grade 35/50 (according to EN 12591), commercially sold by Nynas under the name “Nybit 40” (Table 1).
- A paraffinic bitumen with penetration grade 35/50, commercially sold by TotalEnergies under the name “Azalt 35/50” (Table 1).

Table 1 presents the detailed physical and chemical properties for both bitumens, together with the respective standard test methods. The binders were tested using DSC 1 Mettler Toledo, an advanced differential scanning calorimetry apparatus following the method described in the literature^33^. Naphthenic bitumen exhibited a higher glass transition temperature (Tg = − 16.4 °C), indicating reduced flexibility at low temperatures, whereas paraffinic bitumen with a lower Tg (–20.6 °C) demonstrated greater flexibility under these conditions. Additionally, the crystallizable paraffin content (FC) measured by DSC was found to be 5.2% for paraffinic bitumen, whereas no crystallizable paraffin was detected for naphthenic bitumen. Finally, the carbonyl index obtained via Fourier-transform infrared spectroscopy (FTIR Thermo Scientific Nicolet iS20) calculated according to the method described in reference (56), showed that naphthenic bitumen had a significantly higher carbonyl index (0.0570) compared to paraffinic bitumen (0.0231), thus confirming its higher acidity.

**Table 1:** Properties of naphthenic and paraffinic bitumen.

Property	Standard	Unit		
Bitumen type			Naphthenic	Paraffinic
Penetration Grade	EN 12591	-	35/50	35/50
Dynamic viscosity at 140 °C	EN 12596	Pa.s	497.9	615.5
Density at 140 °C	EN 15326	g.cm^-3^	0.961	0.968
Glass transition	DSC	°C	-16.4	-20.6
Content of crystallizable paraffins (FC)	DSC	%	0	5.2
Carbonyl Index	FTIR	-	0.0570	0.0231

Both 35/50 binders meet the same specification range, yet the naphthenic grade shows a higher glasstransition temperature (–16.4 °C) and twice the carbonyl index, whereas the paraffinic binder contains 5% crystallisable paraffins.

### Fillers

The calcareous filler (F.C) used in this study is a limestone powder sourced from a French white marble quarry belonging to La Provençale in the South of France. It is sold under the name “Mikhart 15”. It’s characterized by high-purity with a concentration of 99.4% CaCO₃. On the other hand, the siliceous filler (F.S) is a quartz powder obtained from the Maisse production site of Fulchiron in the Paris area. This filler is sold under the name S400 and has a high purity of 94.6% silicon dioxide (SiO₂).

The physical properties of the fillers determined using standard methods are listed in Table 2. The fillers were selected to ensure a similar particle size distribution, as detailed in the subsequent sections.

**Table 2:** Physical and chemical properties for both (F.C) and (F.S).

Fillers	Standard	F.C	F.S
Real density (Mg/m^3^)	NF EN 1097-7	2.76	2.74
Loss on ignition 105 °C to 1000 °C (%)	NF EN 15,169	43.80	0.40
pH	NF X31-103	8.66	7.86
Redox potential (mV)	NF ISO 11,271	114.80	166.70
Conductivity κ (µS/cm)	NF EN ISO 11,275	42.10	0.43
Rigden Voids (%)	NF EN 1097-4	30.3	35.2
BET surface area (m²/g)	BET (N_2_ adsorption)	1.30	1.66

Both fillers have similar density and BET area, yet the limestone (F.C) is more alkaline (pH 8.7) and more soluble in water (higher conductivity), while the quartz (F.S) exhibits a higher redox potential, consistent with its Silanol-rich surface.

#### X-ray fluorescence analysis (XRF)

The chemical composition of the fillers was determined using X-Ray fluorescence (XRF) analysis on a Thermo Scientific™ ARL™ PERFORM’X Sequential X-Ray Fluorescence Spectrometer. The samples were prepared by drying and grinding the fillers to a fine powder to ensure homogeneity. Approximately 5 g of each filler were placed in sample cups for analysis. The XRF method provides precise elemental composition by detecting the characteristic X-rays emitted by each element when exposed to a primary X-ray beam. This technique allowed for the quantification of major and minor elements in the fillers (Table 3).

**Table 3:** Chemical composition of (F.C) and (F.S) by X-ray fluorescence.

Chemical element	Oxide equivalent	F.C	F.S
Si	SiO_2_	0.20%	94.61%
Ca	CaCO_3_	99.36%	-
Al	Al_2_O_3_	0.15%	4.09%
Fe	Fe_2_O_3_	-	0.05%
K	K_2_O	-	0.91%
Ti	TiO_2_	-	0.06%
Cl	-	0.14%	0.20%

XRF data demonstrate the fillers’ high purity: F.C is almost pure CaCO_3_ (99.4%), whereas F.S consists of 94.6% SiO_2_ with minor Al_2_O_3_ and K_2_O.

#### X-ray diffraction analysis (XRD)

The characterization of the crystalline phases of F.C and F.S was carried out using X-ray diffraction (XRD). The samples were prepared by grinding the fillers into a fine powder. This powder was then analyzed using an X-ray diffractometer equipped with a CuKα source (λ = 1.54 Å). The scanning was conducted in a 2θ range from 5 to 80 degrees^34,35^.

**Fig. 1:**
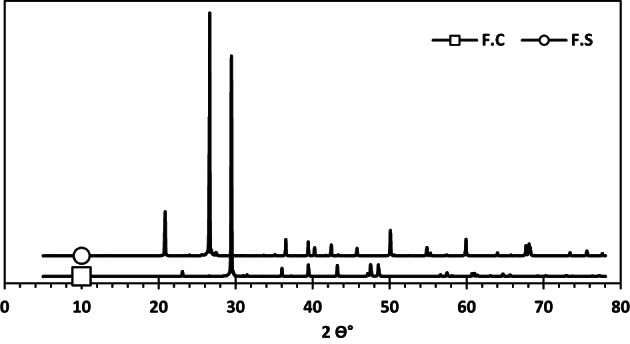
X-ray diffraction (XRD) patterns for F.C and F.S. This diffractogram highlights strong calcite peaks (2θ ≈ 29°, 39°, 47°) for the calcareous filler (F.C), whereas the siliceous filler (F.S) shows quartz reflections at about 21°, 26° and 50°. Traces are vertically offset for clarity.

The resulting diffraction spectra (see Fig. 1) confirmed the dominant mineralogical nature of each filler. The F.S exhibited strong peaks at approximately 21°, 26° and 50° (2θ), characteristic of crystalline SiO₂, indicating its high purity. In contrast, the F.C showed intense peaks near 29°, 39° and 47° (2θ), consistent with calcite (CaCO₃) as the main crystalline phase^36,37^.

#### Laser diffraction (wet dispersion): time-resolved protocol

The granulometric analysis of the F.C and F.S was carried out using a Coulter LS13320 laser diffraction particle size analyzer under wet dispersion modes. The wet method used distilled water as the dispersing medium. Optical settings were selected according to standard recommendations in the literature, particularly the real and imaginary refractive indices (RI and IRI), in accordance with the work of Keck and Müller^38,39^. Additionally, a dedicated wet-route protocol was implemented to assess the tendency of the fillers to agglomerate in aqueous media^14,40^. Series of tests were performed to monitor the size distribution of fillers in aqueous suspension with magnetic mixing at 400 rpm and sampling at 15, 30 and 60 min. Laser diffraction measurements were carried out after each interval to record the particle size distribution over time.

#### Preparation of reference granulometric distributions by optical microscopy

To generate a reference granulometric curve for each filler, a rigorous dispersion protocol was applied using water as a medium. A 5 wt% suspension of either F.C and F.S was mechanically stirred for 30 min at 400 rpm using a magnetic stirrer^41^ followed by ultrasonic treatment at 40 kHz with 90% amplitude for 15 min, maintaining the bath temperature at 25 °C. This process ensured effective deagglomeration through hydrodynamic shear and acoustic cavitation^42^.

**Fig. 2:**
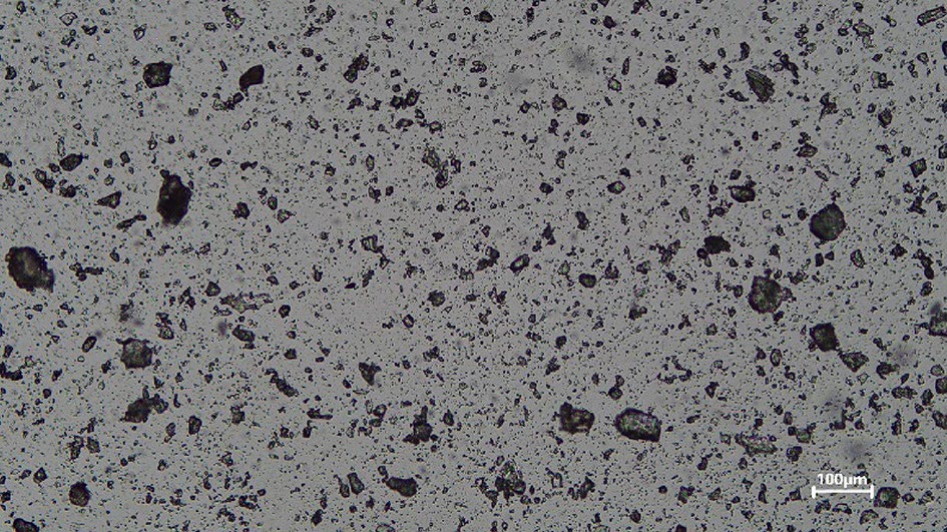
Optical microscope images of F.C: Image with adjusted transmitted light. Transmittedlight micrograph (10× objective, ≈ 1.3 μm resolution): dark grains on a bright background provide optimal contrast for ImageJ segmentation.

A sample was then collected from the beaker and placed between two glass slides for transmitted light microscopy. Imaging was performed with a Leica DM 2500 M microscope fitted with a Plan EPI 10×/0.25 objective (≈ 1.3 μm resolution). The illumination was adjusted so that the particles appeared dark on a bright background (Fig. 2) allowing for clear segmentation using ImageJ. Eight images per filler type were captured from different zones and analyzed through image processing.

### Mastics

#### Preparation of bitumen–filler mastics, microscopic imaging and granulometric analysis via image-based processing

**Fig. 3:**
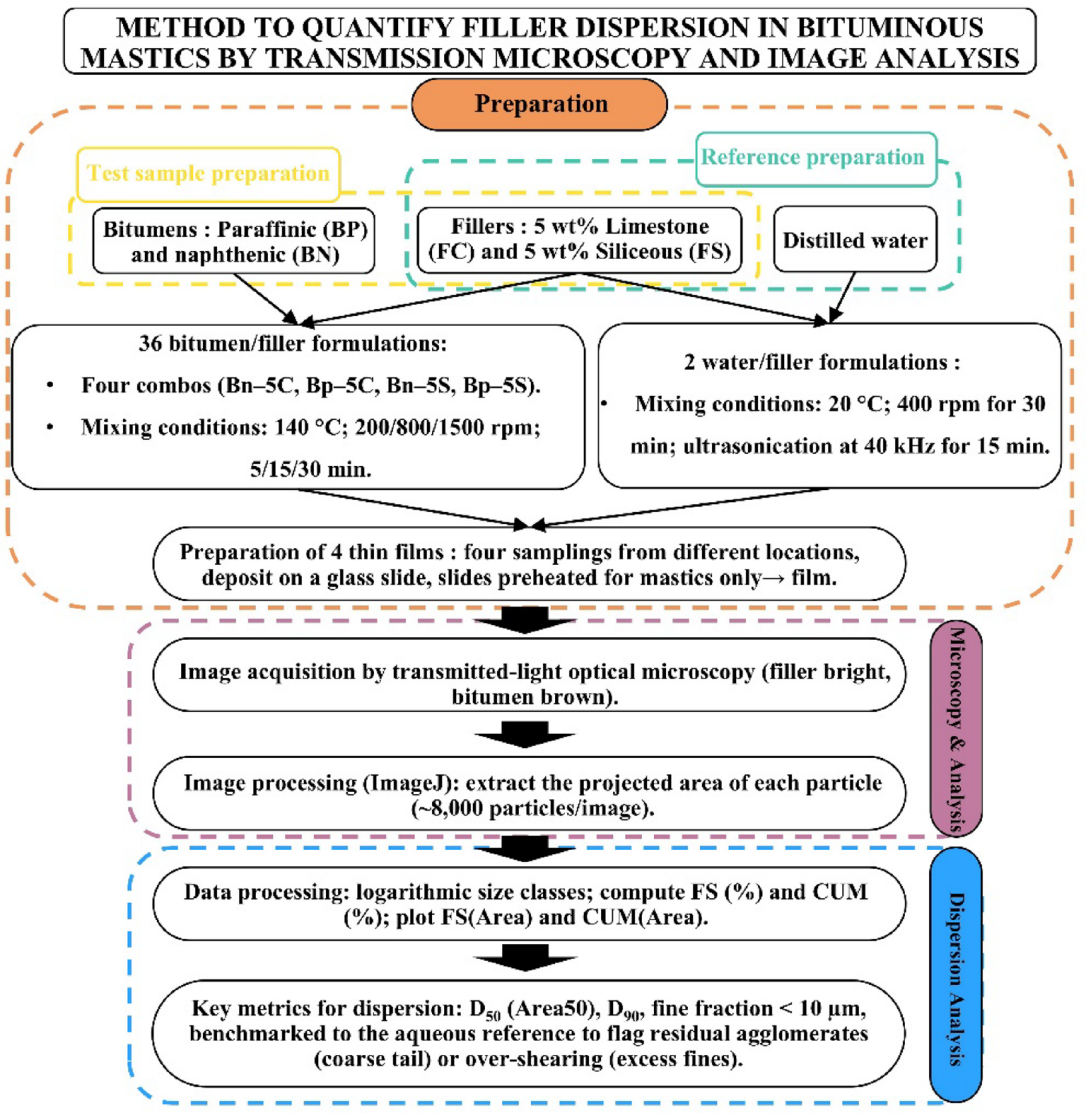
Experimental workflow for quantifying filler dispersion in bituminous mastics. Schematic representation of the procedure from sample preparation to image analysis and dispersion metrics extraction.

Figure 3 summarizes the method used to quantify filler dispersion: four bitumen/filler combinations (Bn–5 C, Bp–5 C, Bn–5 S, Bp–5 S) are mixed under controlled conditions, cast as thin films and imaged by transmitted-light optical microscopy. Images are processed to segment particles and extract projected areas (ImageJ, global auto-threshold algorithms), yielding surface-weighted size distributions plotted in differential (FS) and cumulative (CUM) forms. Key dispersion metrics, median equivalent diameter D_50_ (Area_50_), D_90_ and fine fraction < 10 μm are interpreted against the aqueous, ultrasonically dispersed reference to detect residual agglomerates or over-shearing.

##### Choice of filler concentrations

A filler concentration of 5 wt % was selected for all subsequent tests, as 1 wt % contained too few particles to affect microstructure, while concentrations > 10 wt % produced dispersions that were too dense to observe or control.

##### Preparation of Filler-Bitumen mastics

Thirty-six mastics were compounded at 140 °C in a bladed mixer: naphthenic (Bn) or paraffinic (Bp) bitumen containing 5 wt % limestone (5c) or siliceous (5s) filler, then mixed for 5, 15–30 min at 200, 800 or 1500 rpm. Throughout the manuscript we adopt the shorthand *Bx-5y-V-T*, where *Bx* is the binder type, *5y* the filler nature, *V* the mixing speed and *T* the mixing time.

**Fig. 4:**
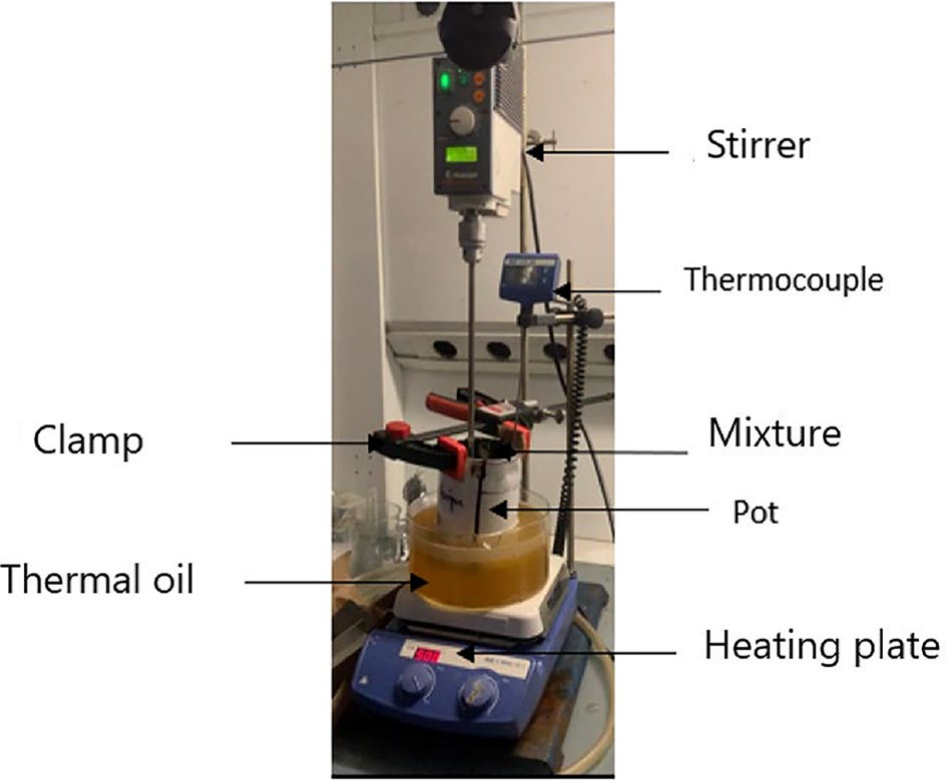
Setup for Bitumen-filler mixing process. Mixing apparatus with precise 140 °C (± 0.5 °C) control and 200–1500 rpm via a four-blade impeller in a thermal oil bath ensuring reproducible energy input for all formulations.

The filler-bitumen blends. also called mastics. were obtained using a specially-developed equipment. shown in Fig. 4. It allowed for precise temperature control at 140 °C ± 0.5 °C using an electronic thermocouple connected to a heating plate. The mixing speed was also controlled at 200. 800 and 1500 rpm during the mixing preparation with a mixer that has a four-blade rod.

Before mixing. 133 g ± 0.1 g of bitumen (representing 95% of the final mastic) is placed in a clean metal container and preheated in a ventilated oven at 140 °C. Simultaneously. 7 g ± 0.01 g of filler (5% of the final mass) is dried in the same oven at 140 °C. After 4 h. the bitumen container is transferred to a thermal oil bath. kept at 140 °C and an agitator is introduced under mixing (200. 800. or 1500 rpm). A thermocouple ensures the bitumen maintains a stable temperature of 140 °C. Five minutes into the mixing. the filler is carefully added in about 5 s to ensure optimal dispersion. Mixing continues for 5. 15–30 min depending on the experimental conditions. The repeatability of this entire process was tested (see Sect. 3.7) and found to be satisfactory.

##### Preparation of bituminous films for optical microscopy

**Fig. 5:**
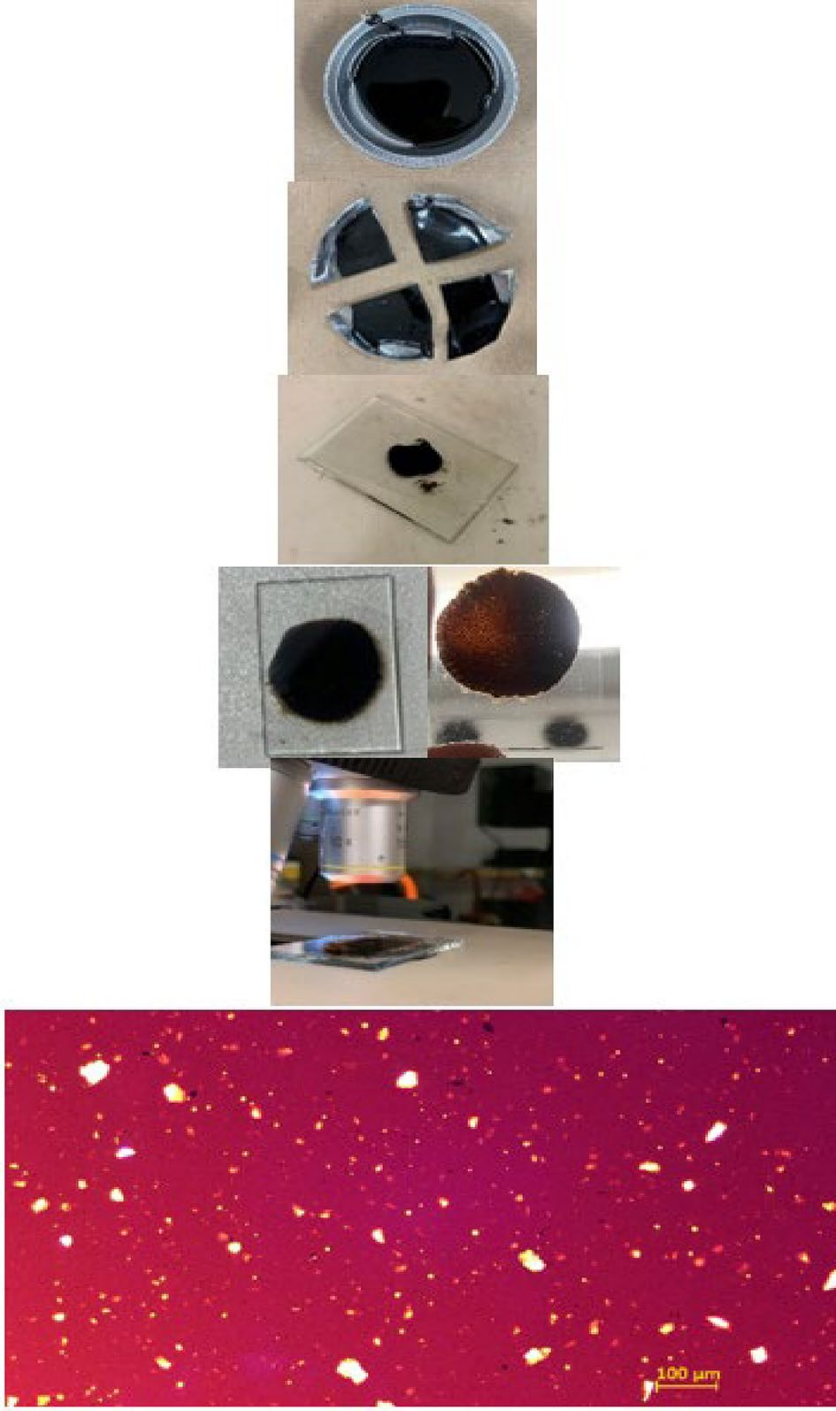
Preparation of bitumen/filler sample for microscopy. A < 110 μm film is spread on a slide at 130 °C and cooled, this thickness allows light transmission and exposes mineral particles in the bitumen.

Each mastic, prepared as described in the previous section, was poured into a metal dish and cooled to room temperature. Four aliquots of 2.00 ± 0.05 mg were sampled, placed on glass slides pre-heated to 130 °C and immediately drawn into thin films under a cover slip. This thickness allows the transmitted light from the microscope stand to pass through the binder and clearly disclose the mineral particles (Fig. 5). Imaging was performed in transmitted-light mode with a Leica DM 2500 M microscope fitted with a Plan EPI 10×/0.25 objective, suitable for the 0–110 μm size range of the fillers. The protocol is simple, low-cost and sufficiently powerful to assess filler dispersion inside bitumen providing a practical alternative to heavier techniques such as SEM.

##### Image segmentation and projected-area extraction

ImageJ, an open-source platform that provides sub-pixel spatial calibration, automated thresholding algorithms and batch particle-analysis routines, was used to quantify and visualise the spatial distribution of filler particles within the bitumen^43^. Micrographs were processed with ImageJ. Calibration used a stage micrometer: 281 pixels equal 100 μm giving 1 px = 0.356 μm. After calibration, binary thresholding (Huang method) separated mineral particles from the binder. For each particle, the software recorded projected area^44^. Each calibrated frame delivers on average ≈ 8,000 individual particles providing a robust statistical basis for subsequent morphometric analysis.

##### Surface-weighted size-distribution construction

To establish the granulometric curve for the filler dispersed in water (reference) and, in the same way for the filler embedded in bitumen, we work directly with the projected areas Ai delivered by ImageJ. Size classes are first defined in terms of diameter on a logarithmic scale

1 $${D_n}={D_0} \times {r^{\left( {n - 1} \right)}}~{\text{~Avec~}}r=~{10^{{\raise0.7ex\hbox{$1$} \!\mathord{\left/ {\vphantom {1 k}}\right.\kern-0pt}\!\lower0.7ex\hbox{$k$}}}}$$

where D_0_​ is the starting diameter and k = 4 classes per decade, leading to *r* ≈ 1.778. Diameter bounds [D_*n*−1_,D_n_​] are then translated into area bounds without altering the raw data:

2 $$A_{n}^{*}=\frac{\pi }{4}D_{n}^{2}$$

Each experimental area Ai ​ is assigned to its corresponding interval [$A_{{n - 1}}^{{\text{*}}}$, $A_{n}^{{\text{*}}}$]. The partial surface of class n is

3 $$~{A_n}=\mathop \sum \limits_{{{A_i}~ \in \left[ {A_{{n - 1}}^{*},~A_{n}^{*}} \right]}} {A_i}$$

d the differential surface fraction is obtained through

4 $$FS=\frac{{{A_n}}}{{\mathop \sum \nolimits_{i} {A_i}}} \times 100~~$$

Cumulative summation of FS yields A_cum_(D). Because the identical processing sequence is applied to both the aqueous suspensions and the bituminous films, the resulting surface-weighted distributions are fully comparable and reveal the impact of large particles and agglomerates. The curve obtained from the optimally dispersed aqueous suspension serves as the benchmark and the curves derived from bitumen samples are evaluated against this benchmark to quantify dispersion efficiency as a function of binder chemistry filler type and mixing conditions.

##### Data presentation and key metrics for dispersion analysis

To rigorously assess filler dispersion in bitumen, we employ complementary granulometric representations and indices. Surface-area-based distribution curves are plotted in both differential form (surface fraction, FS) and cumulative form (A_cum_). The differential FS curves highlight the size ranges where most particle surface area concentrates revealing whether the dispersion remains broad (indicating residual agglomerates) or becomes narrow and unimodal (indicating a uniform fine dispersion). The cumulative curves, on the other hand facilitate extraction of key percentile diameters – notably the median equivalent diameter (D_50_ or Area50) and higher percentiles (e.g. D_90_) – which quantify the shift in particle size during mixing. For instance, tracking the position of D_50_ and how far the 50–100% tail of the cumulative distribution extends allows detection of persistent large clusters or, conversely, over-shearing if the tail moves below the reference (fully dispersed) curve. In addition, we monitor the fine particle fraction (< 10 μm) as a practical indicator of dispersion completeness. In a fully de-agglomerated filler (reference state in water), roughly 90% of the total particle surface lies below ~ 10–11 μm diameter. Thus, the percentage of particles finer than 10 μm in bitumen–filler mixtures provides a direct metric to compare against this benchmark of homogeneity. Using these curves and indices in tandem is crucial because each captures different aspects: the FS and D_50_ reflect the overall shift toward finer sizes while the cumulative tail (D_90_ or % > 10 μm) reveals any residual coarse agglomerates or excessive fragmentation. This approach is applied for both filler types and bitumen types enabling a detailed comparison of mixing effects.

## Results and discussion

### Aqueous dispersion kinetics (laser granulometry- wet Route)

**Fig. 6:**
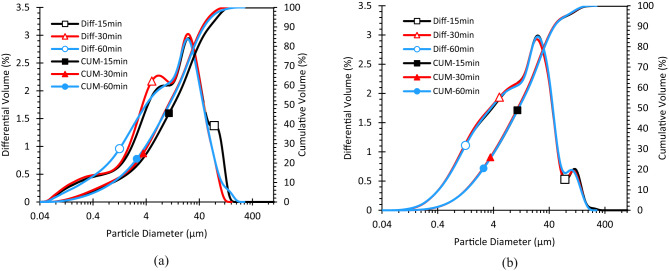
Increase in the particle size distribution of F.C and F.S over mixing time. During wet stirring (400 rpm), the volumetric D_50_ of F.C drops from 13 μm to 10 μm within 30 min, whereas F.S levels off near 11 μm after only 15 min indicating faster deagglomeration of quartz in water.

After 15 min of mixing, the volumetric D_50_ of the calcareous filler (F.C) remains high (13.3 μm, Table 4; Fig. 6), whereas the siliceous filler (F.S) is already close to its steady value (≈ 11 μm). F.C requires ~ 30 min to reach 10.8 μm and then stabilizes, in parallel, its D_90_ decreases from 59 μm to 46 μm (Table 4) indicating gradual agglomerate break-up. This kinetic contrast is consistent with electrokinetics in water, quartz surfaces, rich in silanols, typically carry strongly negative zeta potentials in the neutral alkaline range ( ≈ − 65 mV between pH 7 to 11), which favors electrostatic repulsion and rapid de-agglomeration of F.S suspensions^45^. By contrast, CaCO_2_ surfaces exhibit weaker negative ζ under similar conditions and the availability of Ca^2+^ in solution compresses the diffuse double layer and promotes short range bridging, which slows the early break-up of F.C agglomerates^45,46^. In cement-type aqueous media, limestone fillers further show that surface charge/wettability (together with fineness) govern the flow/dispersion behavior of suspensions, supporting the mechanistic link between ζ, wetting and particle rearrangement observed here (drop in D_90_, plateau of D_50_)^47^. Overall, the laser-granulometry trends (fast approach of F.S to ≈ 11 μm, delayed but monotonic reduction for F.C) align with an electrostatic control scenario in which siliceous particles disperse faster, while calcareous agglomerates require longer mixing to overcome weaker repulsions.

**Table 4:** Volume percentage as a function of F.C and F.S particle diameter over mixing time.

Volume %	F.C			F.S		
	15 min- particle diameter µm	30 min- particle diameter µm	60 min- particle diameter µm	15 min- particle diameter µm	30 min- particle diameter µm	60 min- particle diameter µm
10	0.96	0.94	0.82	1.28	1.27	1.29
25	4.23	3.18	3.53	3.45	3.36	3.45
50	13.27	10.82	10.50	11.34	10.94	11.32
75	31.19	27.28	26.69	28.01	27.38	28.25
90	59.08	48.87	45.68	49.76	48.54	49.85

At 200 rpm the volumetric D_50_ of F.C falls from 13.3 μm to 10.8 μm within 30 min, whereas F.S stabilises near 11 μm after 15 min; the gradual drop of F.C D₉₀ reflects the slower breakup of limestone clusters.

### Setting the reference dispersion curve for the non-aggregated filler

**Fig. 7:**
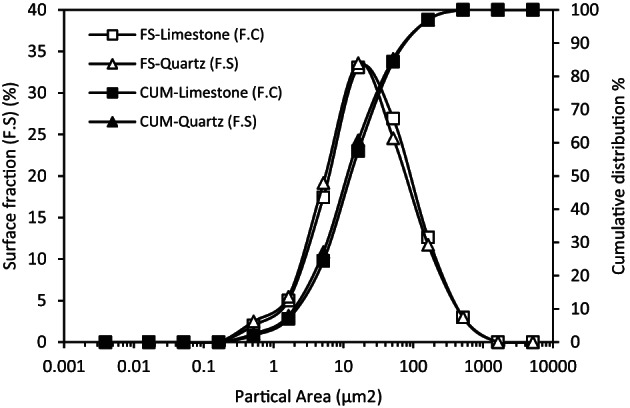
F.C and F.S particle size distribution using optical microscopy. Both fillers dispersed in water display a unimodal peak at 20–30 μm² with 90% of surface below 100 μm^2^, this distribution is taken as the fully dispersed reference.

To establish a baseline dispersion state before analysing behaviour in bitumen, both fillers were fully de-agglomerated in water and characterised by optical microscopy with ImageJ processing. Figure 7 displays the differential surface-fraction (FS) versus individual particle projected area, together with its cumulative curve (CUM). FS-Limestone (F.C) and FS-Quartz (F.S) show nearly overlapping, unimodal profiles centred at 20–30 μm^2^ with 90% of the total surface below 100 μm^2^. These distributions obtained after stringent hydrodynamic and ultrasonic dispersion, constitute the benchmark curves against which all bitumen-based distributions will be compared, any right-ward shift will reveal residual agglomeration.

Optical microscopy with ImageJ allows for direct visualization and measurement of individual particles. By capturing images and analyzing them with specialized software. this approach considers particle shapes and provides a numerical distribution based on surface area. Unlike laser granulometry which relies on theoretical models of light scattering. optical microscopy directly measures the physical characteristics of particles^48^.

### Optical microscopy visualization of agglomerates in both bituminous binders

**Fig. 8:**
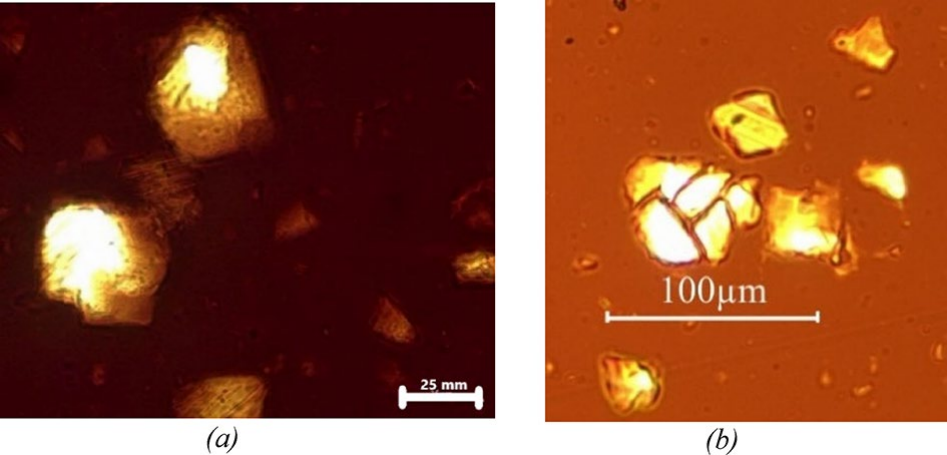
The left image (a) shows the F.C in paraffinic bitumen, while the right image (b) presents the same filler in naphthenic bitumen. The left image (a) displays agglomerated F.C grains in the paraffinic binder, while the right image (b) shows the same type of agglomerates in the naphthenic binder after 5 min of mixing at 200 rpm. The side-by-side view highlights the early formation of filler clusters in both binders, prior to extensive dispersion.

**Fig. 9:**
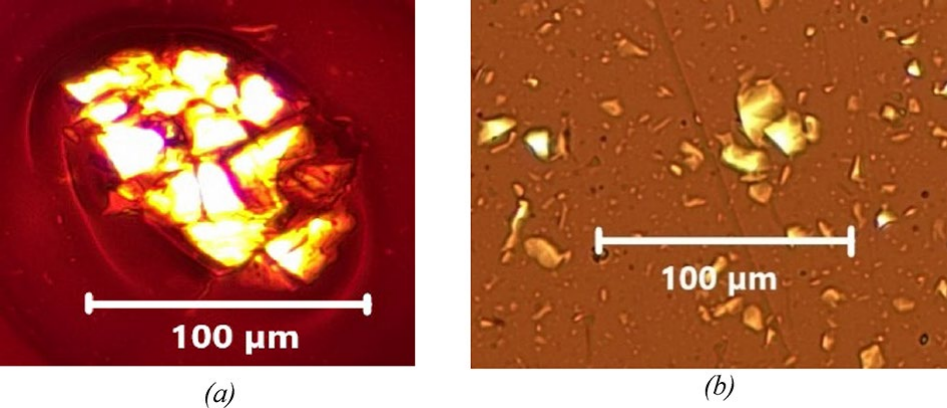
The left image (a) shows the F.S in paraffinic bitumen, while the right image (b) presents the same filler in naphthenic bitumen. The left image (a) displays agglomerated F.S grains in the paraffinic binder, while the right image (b) shows the same agglomerates in the naphthenic binder after 5 min of mixing at 200 rpm.

The images below (Figs. 8 and 9) show the agglomeration of F.C and F.S particles in two types of bitumen. observed under an optical microscope after 5 min of mixing at 200 rpm.

### Study of limestone filler dispersion in two types of bitumen

#### Influence of mixing time on dispersion

**Fig. 10:**
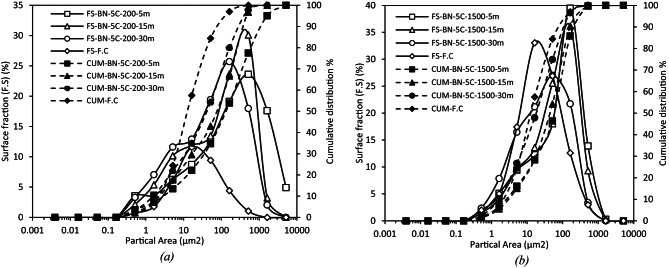
Surface-area-weighted particle-size distributions of F.C dispersed in naphthenic bitumen (5 wt %) as a function of mixing time: (a) low-shear mixing at 200 rpm; (b) high-shear mixing at 1500 rpm. D_50_ drops from 16 μm to 6 μm in 30 min at 1500 rpm, but only to 9 μm at 200 rpm; the cumulative tail confirms faster removal of coarse agglomerates under high mixing.

To better illustrate the impact of mixing energy on filler dispersion, Fig. 10 presents two representative sets of particle-size distribution curves (surface fraction FS and cumulative area CUM vs. projected particle area) derived from optical microscopy and ImageJ analysis. The selected examples correspond to BN-5c-200 and BN-5c-1500 mixtures at three mixing times (5, 15 and 30 min) and are benchmarked against the aqueous reference obtained via optimal water-based dispersion of the F.C. These conditions represent the extremes of low and high shear and provide insight into the combined effects of time and mixing speed on agglomerate breakdown.

At the lowest shear (200 rpm) the median equivalent diameter, D_50_ ​, decreases progressively from about 16 μm after five minutes to roughly 12 μm at fifteen minutes and finally to 9 μm at thirty minutes. Under the highest shear (1500 rpm) the same indicator falls much more rapidly reaching approximately 11 μm within the first five minutes, 9 μm after fifteen minutes and stabilising near 6 μm at thirty minutes. The initial two-thirds reduction observed at 1500 rpm therefore occurs in the time interval during which the 200 rpm mixture is still engaged in breaking only the larger agglomerates^49^.

While the median captures the global shift of the distribution towards smaller sizes, the breadth of the upper tail (D_50_ to D_100_) reveals the persistence of coarse clusters. At 200 rpm, twenty-two per cent of the particle surface area remains above 17.8 μm after five minutes and a non-negligible fraction still extends beyond 100 μm^2^ demonstrating that gentle mixing is initially insufficient to eradicate the largest agglomerates^50^. Although the tail contracts steadily about ten per cent of the surface area is still associated with diameters exceeding 30 μm after half an hour. In contrast, the 1500 rpm protocol suppresses the tail almost completely, less than one per cent of the surface area exceeds 31.6 μm after the first five minutes and by thirty minutes practically the whole distribution is confined below 17.8 μm.

Comparison with the aqueous reference confirms that neither protocol fully recreates the dispersion achieved in water for which ninety per cent of the particle surface lies below roughly 10 μm. Nevertheless, the high-shear route approaches this benchmark far more closely, after thirty minutes seventy-five per cent of the surface is already below 10 μm, whereas the low-shear route reaches only fifty-four per cent. The data therefore suggest a two-regime mechanism, an initial, shear-controlled disintegration of agglomerates followed by a slower, time-dependent refinement of fragments. High shear accelerates the first stage and shortens the second but prolonged exposure beyond fifteen minutes contributes little additional benefit and might even initiate primary-particle breakage, an effect that merits separate mechanical testing but lies outside the scope of the present paper. The paraffinic binder exhibits identical qualitative trends; its curves are not reproduced here for brevity but the numerical values corroborate their equivalence.

#### Influence of shear rate on dispersion

**Fig. 11:**
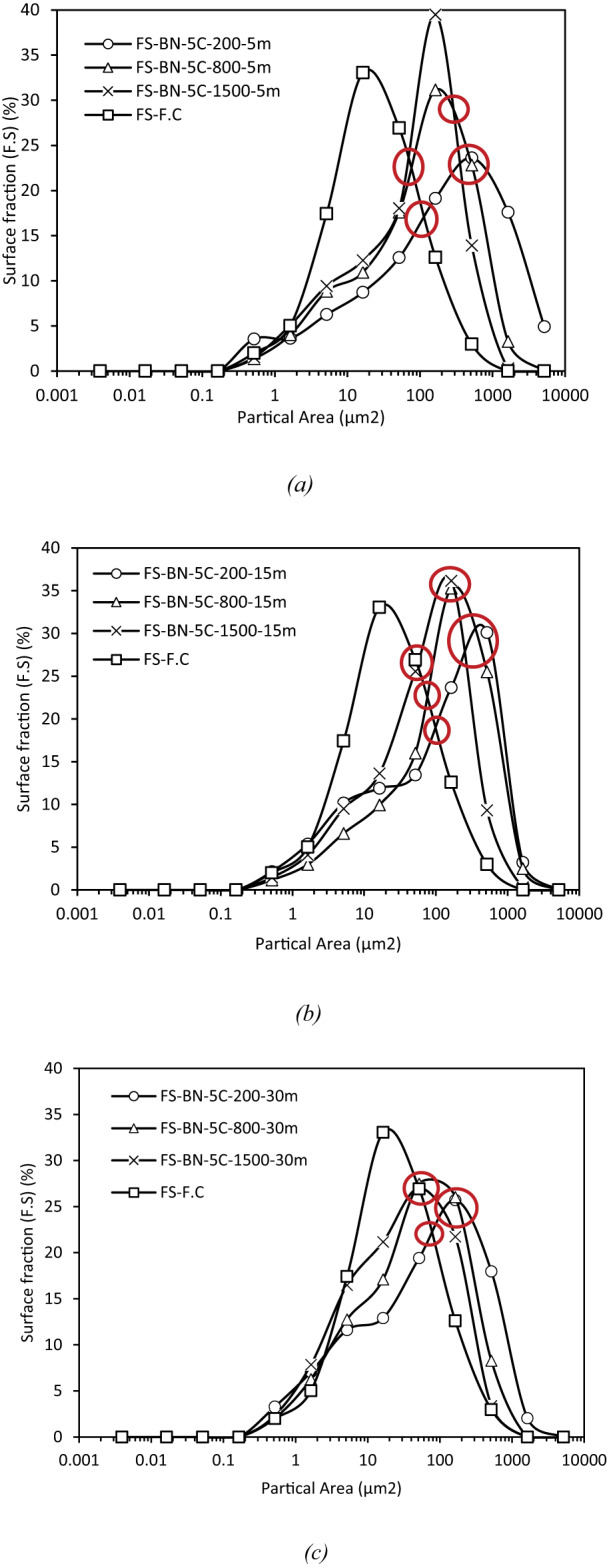
Granulometric distribution curves of naphthenic bitumen with 5% F.C. at mixing speeds of 200, 800 and 1500 rpm. for durations of 5 (a). 15 (b) and 30 min (c). Increasing shear rate and mixing time progressively shifts the modal particle size from around 32 μm^2^ at 200 rpm/5 min to roughly 8 μm^2^ at 1500 rpm/30 min, while narrowing the peak, which denotes a more uniform dispersion.

Figure 11 assembles the differential surface-area distributions obtained after five, fifteen and thirty minutes of mixing at the three angular velocities investigated (200, 800 and 1500 rpm). For any given duration, increasing the impeller speed translates the whole spectrum toward smaller equivalent diameters and simultaneously sharpens the peak attesting to a more efficient disintegration of limestone agglomerates. After five minutes the 200 rpm curve still exhibits its maximum around 550 μm^2^, whereas the 1500 rpm sample already peaks near 200 μm^2^, interpolation of the cumulative data confirms that the median diameter D_50_​ drops from ≈ 16 μm at 200 rpm to ≈ 13 μm at 800 rpm and ≈ 11 μm at 1500 rpm. The same hierarchy persists at fifteen minutes (≈ 14 → 13 → 9 μm) and at thirty minutes (≈ 9 → 7.5 → 6 μm) but the spacing between curves narrows with time, indicating diminishing marginal returns once the coarsest clusters have been dismantled. The upper tail corroborates this saturation: at five minutes about one third of the particle surface in the 200 rpm mixture still lies above 100 μm^2^, compared with 13% at 800 rpm and scarcely 4% at 1500 rpm; after thirty minutes all three tails converge below 300 μm^2^ showing that prolonged mixing can, to a large extent, compensate for moderate shear^51^. Altogether, the data imply that the critical hydrodynamic stress required to rupture the limestone aggregates is already exceeded at ≈ 800 rpm, a further increase to 1500 rpm merely shortens the transient but does not materially alter the equilibrium size distribution reached after half an hour. From a practical standpoint, 800 rpm therefore constitutes an energetically judicious compromise between process intensity and dispersion quality.

#### Influence of type of bitumen on dispersion

**Fig. 12:**
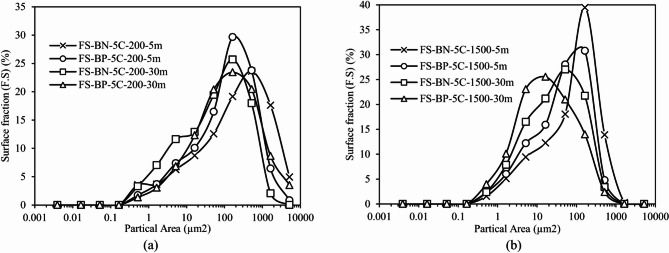
Evolution of the granulometric distribution of F.C in paraffinic and naphthenic bitumen, at two extreme speeds ((a): 200 rpm and (b): 1500 rpm) after 5 and 30 min. At 200 rpm, the paraffinic binder shows a lower D_50_ but the naphthenic one catches up and overtakes after 30 min, at 1500 rpm both binders converge to D_50_ ≈ 6 μm.

Figure 12 juxtaposes the two binders under identical mechanical histories, applying the dual dispersion criteria defined above, namely the median diameter D_50_​ and the breadth of the upper tail (D_50_–D_100_).

At 200 rpm and 5 min (Fig. 12-a), the paraffinic matrix (Bp) displays a lower D_50_ ​ ( 14–15 μm) than the naphthenic one (Bn, ≈ 16 μm) and its differential curve peaks around 200 µm^2^ instead of 500 µm2. Concomitantly, only 28% of the Bp surface area lies above 100 μm², compared with 34% for Bn confirming that early-stage de-agglomeration is faster in Bp under gentle mixing.

After 30 min mixing the situation reverses. The two binders now share an almost identical D_50_ (≈ 9 μm) but the cumulative data reveal that just 2% of the Bn surface area remains beyond 31.6 μm (97.95% already cumulated below that limit), whereas 5.5% of the Bp surface still exceeds it (94.53% cumulated). In other words, the naphthenic binder ultimately produces a shorter tail and therefore a cleaner dispersion while Bp preserves a small fraction of resilient agglomerates. The crossing of the curves highlights a kinetic versus equilibrium contrast: polarity differences favour rapid wetting in Bp, yet the slightly higher viscosity of Bn imposes larger hydrodynamic stresses on the clusters over extended mixing, eventually yielding the finer tail.

At 1500 rpm (Fig. 12-b) the imposed shear overwhelms binder effects. After 5 min both systems converge toward D_50_ ≈ 11 μm and centre their maxima in the 1–3 × 10^2^ µm^2^ band; the surface fraction above 100 μm² drops below 15% in both cases. Prolonging the mixing to 30 min compresses the tails beneath ≈ 2.5 × 10^2^ µm^2^ and drives D_50_ ​ to 6 μm (Bn) and 5–6 μm (Bp), differences that are marginal within experimental uncertainty. Hence, once the hydrodynamic stress comfortably exceeds the cohesive strength of limestone aggregates, chemical interactions that distinguish the two binders govern only the transient kinetics not the final dispersion state.

#### Kinetics of the < 10 μm fraction: joint influence of time and shear

**Fig. 13:**
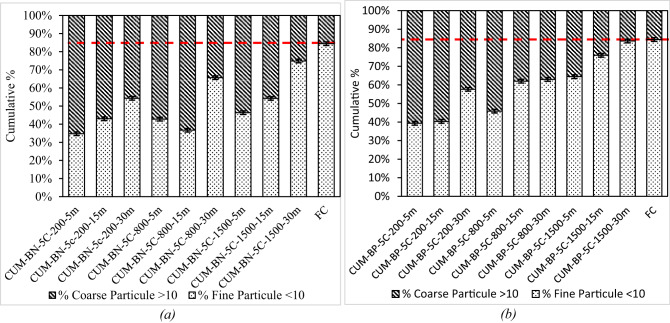
Distribution of fine particles (< 10 μm) and coarse particles (> 10 μm) for different F.C-naphthenic (a) and paraffinic (b) bitumen mixtures. at various mixing speeds and durations. compared to the reference CUM-F.C. The bar charts overlay the cumulative surface share < 10 μm (dotted) and > 10 μm (hatched). The horizontal line at 84.4% represents the fully de‑agglomerated aqueous reference.

**Fig. 14:**
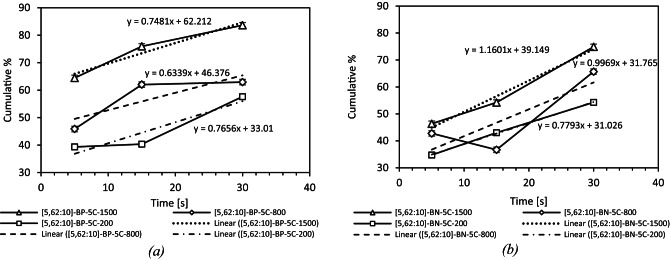
Cumulative [5.62:10] distribution over time for paraffinic (a) and naphthenic (b) bitumen at 200, 800 and 1500 rpm mixing speeds, comparing linear trends. Trend lines reveal faster fine growth in naphthenic binder under high mixing, confirming its sensitivity to mixing energy.

Figures 13 and 14 summarise for each binder, the cumulative surface share of particles finer than 10 μm (dotted bars) and coarser than 10 μm (hatched bars). The horizontal dashed line is placed at 84.4%, the value measured for F.C dispersed in water (class [5.62–10 μm]), approaching this line therefore signals that practically all agglomerates have been broken.

At 200 rpm the paraffinic binder (Bp) already contains ≈ 40% fines after five minutes whereas the naphthenic binder (Bn) starts closer to 33%. Linear fits to the three time-points yield slopes of 0.77% min^–1^ for Bp and 0.78% min^–1^ for Bn, the similar kinetics combined with the higher intercept of Bp, explain why Bp keeps a small but persistent lead up to 30 min. Yet both systems remain far below the 84.4% benchmark indicating that gentle mixing alone cannot reproduce the aqueous dispersion within half an hour.

Raising the speed to 800 rpm accelerates the build-up of fines. The slopes increase to 0.64% min^–1^ for Bp and 1.00% min^–1^ for Bn. Because the naphthenic curve is steeper, the initial gap closes: after 30 min Bp reaches ≈ 70% fines, Bn ≈ 66%. The coarser tail (> 10 μm) shrinks accordingly but a residual 15–20% of the surface area still lies above the threshold emphasising that moderate shear cannot completely eradicate the largest clusters.

Under high shear (1500 rpm) mechanical stress dominates. The fine fraction jumps to ≈ 60% (Bp) and ≈ 55% (Bn) after five minutes, then grows with slopes of 0.75% min^–1^ (Bp) and 1.16% min^–1^ (Bn). By 30 min both binders attain 80–83%—within ± 1% of experimental scatter from the reference line—and their coarse fractions collapse below 17%. The markedly higher slopes for Bn at 800 and 1500 rpm confirm that naphthenic bitumen is more sensitive to mixing conditions: although it lags behind at early times, it benefits more from extended mixing and high shear, eventually matching or surpassing Bp.

### Study of quartz filler dispersion in two types of bitumen

#### Influence of mixing and shear rate time on dispersion

**Fig. 15:**
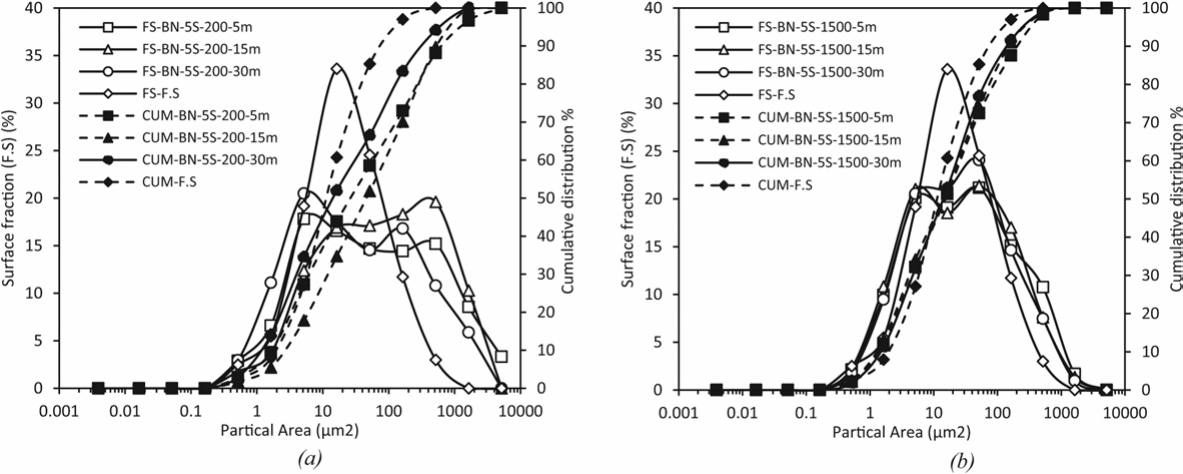
Evolution of the granulometric distribution (differential and cumulative volume) of F.S in naphthenic bitumen (a)- at 200 rpm and (b)- at 1500 rpm after 5, 15 and 30 min, compared to the reference F.S. At 200 rpm the spectrum remains bimodal; the main peak shifts from ≈ 14 μm to 11 μm, yet ~ 6% of the cumulative surface still lies above 31 μm^2^ indicating residual cohesive quartz clusters. At 1500 rpm the distribution is already narrow (8–12 μm^2^) after 5 min, D_50_ levels off near 4 μm and the < 10 μm share increases only slightly from 72–77%, confirming that most agglomerates break up during the very first minutes of high-energy mixing.

The selected examples correspond to BN-5 S-200 and BN-5 S-1500 mixtures examined at three mixing times (5, 15 and 30 min) and benchmarked against the aqueous reference obtained via optimal water-based dispersion of the F.S. Figure 15 juxtaposes two boundary shear protocols applied to the same naphthenic mastic. At 200 rpm (Fig. 15-a) the differential spectrum remains bimodal throughout the test, with a broad maximum shifting from ∼14 μm to ∼11 μm equivalent diameter as mixing proceeds from 5 to 30 min. The cumulative curve shows that the fine fraction (< 10 μm) rises from 58% after 5 min to 67% after 30 min while the median diameter D_50_​ migrates from ≈ 7.5 μm to ≈ 6 μm. The upper tail (D_50_​–D_100_​) still contains ≈ 6% of the total surface at diameters above 31.6 μm indicating a residual cohort of cohesive clusters.

Under 1500 rpm (Fig. 15-b) the initial spectrum is already narrow and centred on projected areas of 8–12 μm^2^; D_50_​ lies near 4 μm after the first 5 min and remains essentially unchanged thereafter. The cumulative fraction of fines increases only slightly, from 72 to 77%, between 5 min and 30 min and the coarse tail collapses below 4%. Hence, most quartz agglomerates are dismantled during the very first minutes when the hydrodynamic stress is highest, subsequent mixing merely polishes the distribution^52^.

#### Kinetics of the fine fraction and binder effect

**Fig. 16:**
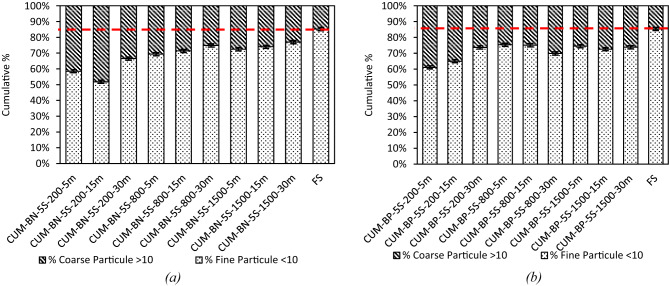
Distribution of fine particles (< 10 μm) and coarse particles (> 10 μm) for different F.S-bitumen mixtures (naphthenic and paraffinic) at various mixing speeds and durations. compared to the reference CUM-F.S. Whatever the mixing route, the < 10 μm share grows only modestly: from 58–61% to 67–70% at 200 rpm, and from 72–74% to 76–77% at 1500 rpm. Linear slopes below 0.3% min⁻¹ an order of magnitude lower than for F.C show that quartz deagglomeration plateaus rapidly; extended mixing merely trims the coarse tail, which never falls below ~ 5% of the total surface indicating that ultimate dispersion is limited by intrinsic aggregate cohesion rather than hydrodynamic conditions.

Figure 16 condenses the cumulative data into the two dispersion indices previously retained. Whatever the mixing protocol, the increase of the fine share is modest. At 200 rpm the proportion of particles < 10 μm grows from 58 to 61% at 5 min to 67–70% at 30 min, at 1500 rpm it passes from 72 to 74% to 76–77% over the same period. Linear fits through the three time points yield slopes not exceeding 0.3% min⁻¹ an order of magnitude lower than those observed for F.C. Dispersion therefore plateaus almost immediately after wetting and prolonged mixing merely trims the residual tail.

Hence, the paraffinic mixtures invariably start from a slightly higher fine-particle baseline, reflecting more efficient initial wetting, nevertheless, additional shear and/or time benefits the naphthenic binder to such an extent that, by the 30-minute mark, the two media become virtually indistinguishable within the ± 1% experimental scatter. In neither binder does the residual coarse fraction fall below about 5% indicating that the ultimate dispersion limit is set by the intrinsic cohesion of the quartz aggregates rather than by hydrodynamic conditions or binder polarity.

### Effect of shear-induced fragmentation on filler particles in naphthenic bitumen at high mixing speeds

**Fig. 17:**
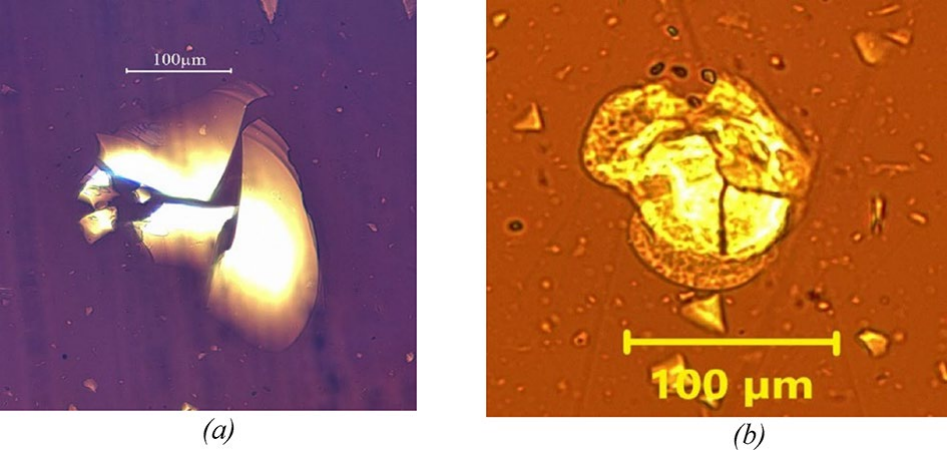
Optical microscopy images showing the effect of 1500 rpm mixing on filler particles in naphthenic bitumen. (a) F.S displaying agglomeration and potential fragmentation; (b) F.C with visible particle break. Intensive mixing at 1500 rpm causes particle break‑up: in image (a) for F.S, cracked grains appear alongside a few remaining clusters, whereas image (b) for F.C clearly exhibits a split limestone particle. These micrographs demonstrate that high mixing energy can mechanically fracture both fillers with fragmentation being more pronounced for F.C.

The images below Fig. 17 illustrate the effects of high-speed shearing (1500 rpm) on the dispersion of fillers in naphthenic bitumen with F.C and F.S. As seen in the image on the left (F.C at 1500 rpm). a clear fragmentation phenomenon is observed with a broken limestone particle. This behavior supports the hypothesis that high mixing speeds can induce excessive shearing leading to the breakage of larger particles into finer ones. This phenomenon contributes to an increase in the cumulative fine particles (< 10 μm). sometimes exceeding the reference. In the image on the right (F.S at 1500 rpm). particle agglomeration is also noticeable suggesting that dispersion is influenced by the nature of the filler and the mixing conditions. This fragmentation. combined with the agglomeration observed under certain conditions. confirms that particle dispersion can be significantly affected by mixing speed. especially in systems involving rigid particles like limestone and quartz.

### Repeatability

All measurements were performed under metrological control across the entire experimental set, weighing on a calibrated balance with a target mass of 5.00 ± 0.1 g per weighing, mixing temperature controlled and logged at 140 °C ± 1 °C using a Pt100 probe, mixing time timed (1-s resolution) with an operational tolerance of ± 30 s to cover handling, fillers pre-conditioned by drying to constant mass, imaging with scale calibration using a stage micrometer and fixed settings (resolution, illumination) in line with good practice for image-based particle sizing, laboratory mixing performed according to the asphalt laboratory mixing standard.

**Fig. 18:**
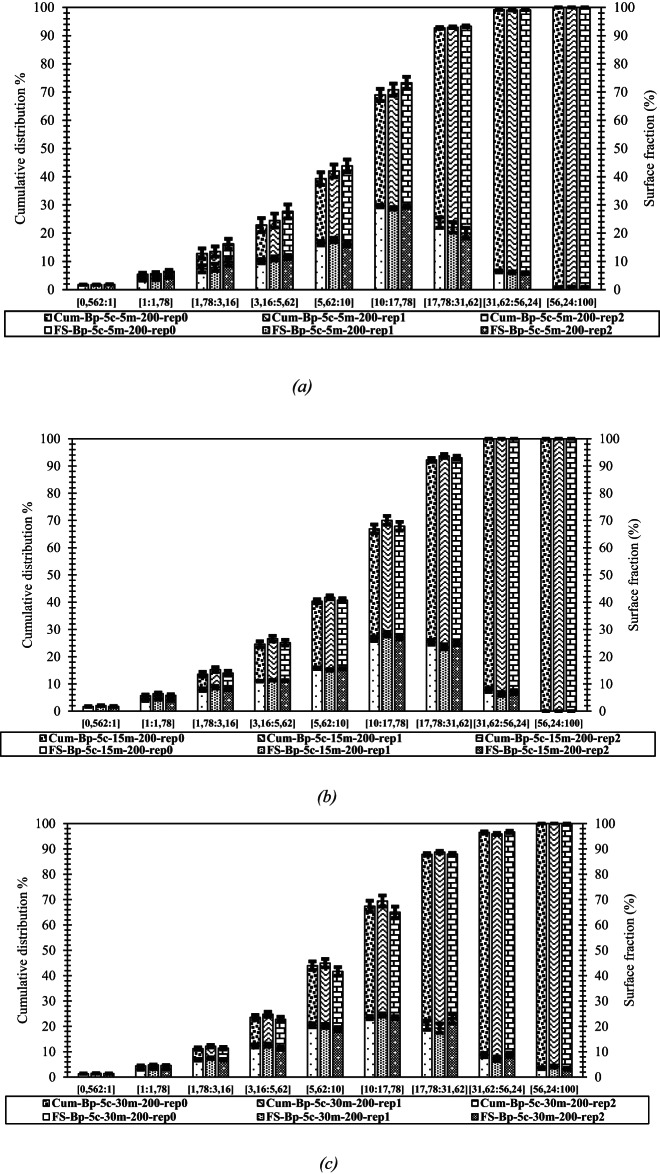
Repeatability of F.C-paraffinic bitumen mixtures at 200 rpm after 5 min (a), 15 min (b) and 30 min (c) with cumulative and surface fraction distributions for three repetitions. The three replicates deviate by < 1% on the [5.62–10 μm] share, demonstrating the robustness of the optical protocol.

To evaluate the repeatability of our area-based method. we performed three different formulations of paraffinic bitumen (Bp) with 5% F.C at 200 rpm for mixing times of 5. 15 and 30 min (see Figs. 18; Table 5). We focused on the [5.62:10] µm size range which contains most particles at 15 and 30 min and omitted 5 min because the blend is not yet fully homogenized. Within this interval. the standard deviation increases from about 0.3 at 15 min to 0.81 at 30 min. to be cautious. we thus apply a ± 1% margin when interpreting our results. This variability partly reflects how sensitive the fine fraction is to the sampling location emphasizing the need to allow sufficient time (at least 15 min) for the filler to be evenly dispersed before conducting granulometric measurements.

**Table 5:** Standard deviations of surface fraction and cumulative volumes for F.C-paraffinic bitumen mixtures at 200 rpm after 5. 15 and 30 min.

Particle diameter range µm	Particle area range µm^2^	5 min		15 min		30 min	
		Standard deviation -FS	Standard deviation -CUM	Standard deviation - FS	Standard deviation -CUM	Standard deviation -FS	Standard deviation -CUM
[0.562:1]	[0.248:0.8]	0.06	0.06	0.16	0.16	0.06	0.06
[1:1.78]	[0.8:2.5]	0.49	0.54	0.26	0.42	0.13	0.18
[1.78:3.16]	[2.5:7.9]	1.25	1.79	0.47	0.88	0.34	0.51
[3.16:5.62]	[7.9:24.8]	0.73	2.46	0.17	1.05	0.63	1.00
[5.62:10]	[24.8:78.5]	0.76	2.26	0.30	0.74	0.81	1.67
[10:17.78]	[78.5:248]	0.45	2.16	0.86	1.60	0.63	2.20
[17.78:31.62]	[248:785]	1.87	0.29	0.92	0.72	1.85	0.49
[31.62:56.24]	[785:2484]	0.33	0.08	0.72	0.00	0.89	0.42
[56.24:100]	[2484:7854]	0.08	0.00	0.00	0.00	0.42	0.00

Across different particle size intervals. These values demonstrate that, under the test conditions (200 rpm, 140 °C), the measurement protocol delivers excellent repeatability; the slight increase to 0.81% at 30 min, confirming the overall reliability of the test.

#### Repeatability and test–retest agreement (Bland–Altman)

Repeatability was assessed on three independent repeats under the same condition (Bp + 5% F.C, 200 rpm) at 5/15/30 min analysing the three pairs: (rep0–rep1), (rep0–rep2), (rep1–rep2) for two image-based metrics: FS [5.62–10] µm (surface fraction within the class) and CUM < 10 μm (cumulative surface fraction). Test–retest agreement was quantified using the Bland–Altman approach (difference vs. mean), reporting bias, SD of differences, and the 95% Limits of Agreement (LoA = bias ± 1.96 × SD) according to the original method and interpretation guidance. Analyses were performed in MedCalc (*Statistics → Method comparison → Bland–Altman plot*), which also provides 95% CIs for bias and LoA based on the t-distribution for small samples.

Table 6 (a) FS [5.62–10] µm and (b) Cum < 10 μm (*n* = 3 pairs per condition) summarise the outcomes. For FS [5.62–10] µm, LoA are tight at 15 min (bias + 0.12, SD 0.51, [− 0.88; +1.12]) and remain moderate at 30 min (bias + 1.00, SD 0.68, [− 0.34; +2.34]); they are wider at 5 min (bias + 0.23, SD 1.29, [− 2.31; +2.76]), consistent with early, non-stabilised mixing. For Cum < 10 μm, agreement is acceptable at 15 min (bias − 0.26, SD 1.25, [− 2.71; +2.19]) and widens at 30 min (bias + 1.50, SD 2.24, [− 2.89; +5.89]), reflecting sensitivity to the 10 μm threshold, at 5 min, the negative bias (bias − 2.99, SD 1.38, [− 5.69; −0.29]) indicates a still-limited fine fraction. Overall, the method shows sufficient metrological stability to track dispersion with FS [5.62–10] µm as the primary metric and CUM < 10 μm as a global trend metric.

**Table 6:** Repeatability and agreement (Bland–Altman), (a) FS [5.62–10] µm and (b) Cumulative < 10 μm at 5/15/30 min (Bp–5 C–200 rpm).

(a)			
	FS [5,62:10]—Bp–5 C-200-5	FS [5,62:10]—Bp–5 C-200-15	FS [5,62:10]—Bp–5 C-200-30
Sample size	3	3	3
Arithmetic mean (Bias)	0.23	0.12	1.00
SD of differences	1.29	0.51	0.68
[Lower: Upper] 95% Limit of Agreement	[− 2.31: +2.76]	[− 0.88: +1.12]	[− 0.34: +2.34]

(b)			
	CUM < 10 μm —BP–5 C-200-5	CUM < 10 μm —BP–5 C-200-15	CUM < 10 μm —BP–5 C-200-30
Sample size	3	3	3
Arithmetic mean	-2.99	-0.26	1.50
SD of differences	1.38	1.25	2.24
[Lower: Upper] 95% Limit of Agreement	[− 5.69: −0.29]	[− 2.71: +2.19]	[− 2.89: +5.89]

Summary of bias, standard deviation, and 95% limits of agreement for replicate image-based dispersion metrics.

## Conclusion

This study proposes a fast and reproducible opticalmicroscopy protocol, coupled with digital image analysis, to quantify the dispersion of mineral fillers in bitumen. The key step is the definition for every filler of a fully dispersed aqueous reference produced by 30 min mechanical stirring (400 rpm) followed by 15 min ultrasonication (40 kHz, 90% amplitude). Comparing surfacebased particlesize distributions in bitumen with this benchmark using the equivalentdisk approach to derive the surfacemedian diameter D_50_ and the cumulative tail up to D_100_—reveals the true state of homogenisation. Results show that at 200 rpm at least 15 min are required to reach a stable dispersion, while 1500 rpm induces overshearing and fragment releases that artificially increase the ultrafine fraction. The method discriminates clearly between a highly hydroxylated quartz, which attains its reference state within five minutes, and an alkaline limestone, whose D_50_ and tail shorten gradually over thirty minutes, the trend matches laserdiffraction data obtained in water confirming the robustness of the optical protocol whose repeatability via a Bland–Altman analysis. Under our conditions (paraffinic 35/50 + 5 wt% limestone, 200 rpm), FS[5.62–10] showed tight limits of agreement at 15 min ( ≈ − 0.9 to + 1.1 pp), whereas CUM < 10 μm broadened up to ≈ − 2.9 to + 5.9 pp at 30 min. By capturing the initial advantage of the paraffinic binder and the subsequent convergence of the naphthenic binder under prolonged mixing, the method highlights the interdependence of filler chemistry, binder polarity and mechanical energy. It therefore furnishes a practical tool for designing mastics with superior homogeneity and durability and opens future work lines dedicated to correlating dispersion trajectories with surfaceenergy parameters and nanoscopic adhesion mapping.

Linking the dispersion trajectories to the binder’s effective polarity, through high-temperature SARA analysis, interfacial-tension measurements and contact-angle tests and to the fillers’ surface chemistry using colloidal-probe AFM, XPS and ToF-SIMS will make it possible to connect adhesion energy with mixing conditions, thereby paving the way for predictive discrete-element or molecular simulations.

## Data Availability

Data sets generated during the current study are available from the corresponding author on reasonable request.
